# Diagnosis, treatment, and prevention of severe acute hepatitis of unknown etiology in children

**DOI:** 10.1007/s12519-022-00581-x

**Published:** 2022-06-30

**Authors:** Ying-Hu Chen, Jin-Gan Lou, Zi-Hao Yang, Qing-Jiang Chen, Chun-Zhen Hua, Sheng Ye, Chen-Mei Zhang, Jie Chen, Zong-Wei Huang, Jin-Dan Yu, Zhi-Gang Gao, Qiang Shu

**Affiliations:** 1 Infectious Disease Department, National Clinical Research Center for Child Health, Children’s Hospital, Zhejiang University School of Medicine, Hangzhou, 310052 China; 2 Gastroenterology Department, National Clinical Research Center for Child Health, Children’s Hospital, Zhejiang University School of Medicine, Hangzhou, 310052 China; 3 PICU, National Clinical Research Center for Child Health, Children’s Hospital, Zhejiang University School of Medicine, Hangzhou, 310052 China; 4 General Surgery Department, National Clinical Research Center for Child Health, Children’s Hospital, Zhejiang University School of Medicine, Binsheng Road 3333, Hangzhou, 310052 China

**Keywords:** Acute Hepatitis, Diagnosis, Epidemiology, Pediatrics, Treatment

## Abstract

**Background:**

Severe acute hepatitis of unknown etiology in children has recently exhibited a global trend of concentrated occurrence. This review aimed to summarize the current available information regarding the outbreak of severe acute hepatitis and introduce our hospital’s previous experiences with the diagnosis and treatment of severe acute hepatitis for reference.

**Data sources:**

Websites including the UK Health Security Agency, European Centre for Disease Prevention and Control, CDC, WHO, and databases including PubMed/Medline, Cochrane Library, Embase and Web of Science were searched for articles on severe acute hepatitis in children.

**Results:**

As of May 26, 2022, a total of 650 cases have been reported in 33 countries; at least 38 (6%) children required liver transplantation, and nine (1%) died. Cases are predominantly aged between 3 and 5 years old, and there are no epidemiological links among them. The common manifestations are jaundice, vomiting and pale stools. Adenovirus tested positive in most cases, and SARS-CoV-2 and other viruses were detected in a few cases, but virus particles were not found in liver tissue. Adenovirus immunohistochemistry showed immunoreactivity in the intrasinusoidal lumen from some liver samples. The hierarchical treatment includes symptomatic and supportive therapy, management of coagulation disorders and hepatic encephalopathy, artificial liver support, and liver transplantation (approximately 6%–10% of cases require liver transplant).

**Conclusions:**

The etiology of this severe acute hepatitis in children is not clear. The clinical features are severe acute hepatitis with significantly elevated liver enzymes. Clinicians need to be alert to children with hepatitis.

## Introduction

In early April 2022, Marsh et al. reported five children with severe acute hepatitis of unknown etiology who were admitted to the Royal Hospital for Children, Glasgow, Scotland within March 2022 [1]. The number of cases has exceeded that of the whole year in previous years [1]. Meanwhile, 9 cases were retrospectively identified from October 2021 to February 2022 at a children’s hospital in Alabama, USA [2]. As of April 18, 2022, the number of cases reported by 6 of the 24 countries around the world is at least 3 times higher than the annual average number of cases in the previous 5 years [3]. On April 23, the WHO reported 169 cases in Europe, America and Asia; 17 cases (10%) needed liver transplantation (LT), and at least one patient died [4]. As of May 26, 650 cases have been reported in 33 countries [5], including 222 cases in Britain, 216 in the USA, 31 in Japan, 29 in Spain, 27 in Italy, 14 in Belgium, 14 in the Netherlands, and 12 in Israel. At least 38 (6%) children have required transplants, and nine (1%) deaths. No cases were reported in the Mainland of China until June 2, 2022.

The UK Health Security Agency (UKHSA), European Centre for Disease Prevention and Control, CDC and WHO have issued a warning and provided case definitions, investigation methods, reporting systems, prevention and control advice [3, 4, 6–8]. This review summarizes the current available information regarding the outbreak of severe acute hepatitis and introduces our hospital’s previous experiences with the diagnosis and treatment of severe acute hepatitis for reference.

## Epidemiological characteristics of reported cases

The reported cases have the following epidemiological characteristics [1, 5–7, 9]: (1) children who are healthy previously, aged from 1 month to 16 years old (predominantly 3–5 years), and mainly of White ethnicity; (2) no epidemiological link except for the first reported cases had a history of close contact; (3) no association with travel, family structure, parents' occupation, diet, water sources, potential exposure to animals or toxicants, or immunosuppression status, and (4) not related to COVID-19 vaccination.

## Hypothesized etiologies

The etiologies of the current outbreak of severe acute hepatitis are still unknown and under investigation. All etiologies reported by the literature and listed here are still hypothesized and need to be further comprehensively studied.

### Adenovirus infection

UKHSA indicated the leading hypotheses remaining to be involved in adenovirus. However, researchers are still continuing to investigate the potential role of other viruses [7]. Nucleic acids or adenovirus antibodies were detectable in most cases. UKHSA reported that adenovirus was detected in 75% of all 131 patients, and 27 (77%) of 35 cases were  infected with adenovirus 41F [9]. Adenovirus 41F usually causes gastrointestinal infection with self-limited infection in children. Disseminated infection complicated with liver inflammation rarely occurs. Some serotypes of adenovirus can cause hepatitis in patients who have received LT or bone marrow transplantation or had ongoing chemotherapy for malignant tumors [10–12]. Adenovirus immunohistochemistry has shown immunoreactivity in the intrasinusoidal lumen from liver samples [7], indicating that immunologic injury induced by adenovirus might be associated with this disease. A novel variant adenovirus with abnormal susceptibility to the liver might be considered to cause viral hepatitis directly [7]; however, no adenovirus particles in the liver tissue were found [2].

### SARS-CoV-2 infection

This outbreak occurred during the COVID-19 pandemic, and more patients were found in countries with a high burden of Omicron variant infection [13]. UKHSA reported that more than 10% of patients had SARS-CoV-2 infection on admission or prior to admission [9]. SARS-CoV-2 superantigen was supposed to be a causal mechanism of multisystem inflammatory syndrome in children [14, 15]. Both SARS-CoV-2 and adenovirus were detected in a few patients [7], and in these patients, adenovirus infection in the intestine might induce a SARS-CoV-2 superantigen reaction and cause immunopathological hepatitis [16].

### **Other virus or multivirus infection**

Other viruses, occasionally two kinds of viruses, were detected in a few patients [7]. Based on metagenomic sequencing, adeno-associated virus 2 (AAV2) was detected in liver tissue or other samples collected from patients in several laboratories. UKHSA reported that when 19 specimens from 11 patients were detected, 9 samples from 8 patients had positive results for AAV2 [7, 9]. There are other possibilities that an unknown virus, or more than one kind of virus, may have infected the host and caused this disease [7, 9].

### Others

A drug, toxin or environmental exposure should be investigated and ruled out [7, 9].

## Clinical manifestations of reported cases

The clinical manifestations of reported cases are acute hepatitis with markedly increased aspartate transaminase (AST > 500 U/L) or alanine transaminase (ALT > 500 U/L). The disease tends to progress rapidly. The most common manifestations are jaundice (71%), vomiting (63%), and pale stools (50%). Most patients have gastrointestinal symptoms in the early stages, including diarrhea (45%), abdominal pain (42%), and nausea (31%). Some present with fever (31%) and respiratory symptoms (19%) [1, 3]. Hepatomegaly is common, but splenomegaly is rare. Hepatic encephalopathy may occur on admission [2].

## Diagnosis

Based on the characteristics of the reported cases and our experiences in managing hepatitis in children. We recommend using the following modalities for diagnosis.

### Laboratory tests

Whole blood, serum, respiratory secretions, feces, urine or other body fluid samples can be sampled according to pathogens, and metagenomic sequencing can be performed if available.

Blood ALT, AST, total bilirubin, direct bilirubin, albumin, glutamyltransferase, prothrombin time, activated partial thrombin time, international standardized ratio (INR), fibrinogen and serum ammonia might be tested to assess the severity. C-reactive protein, procalcitonin, blood gas electrolytes, cytokines, serum ferritin, blood glucose, renal function and myocardial enzymes are helpful for evaluating related complications [2, 17].

Hepatitis viruses A, B, C, D and E, adenovirus, Epstein–Barr virus, cytomegalovirus, SARS-CoV-2, enterovirus, human herpesvirus-6 and 7, parvovirus B19, herpes simplex virus types 1 and 2, etc. Bacteria: *Brucella* spp, *Bartonella henselae*, *Borrelia burgdorferi*, etc. Other pathogenic microorganisms included Mycoplasma pneumoniae, Chlamydia, Leptospirosis, Plasmodium, and Entamoeba. Anti-nuclear antibodies, autoimmune hepatitis antibodies, gamma globulins, etc.

Serum ceruloplasmin, blood genetic metabolic spectrum screening, urine organic acid, etc., and whole-exome and mitochondrial gene examination are feasible if available to exclude inborn errors of metabolism-related liver injury. Blood and urine samples can be analyzed by mass spectrometry to search for possible toxic substances.

Hematoxylin and eosin staining of liver samples, adenovirus immunohistochemistry, Epstein-bar encoding region in situ hybridization, PCR or metagenomic sequencing can be performed.

### Imaging examinations

Abdominal ultrasonography and magnetic resonance imaging (MRI) examinations are performed to assess liver profile, size, and bile duct structure and to identify liver and vascular anatomy and abnormalities in preparation for LT. For patients with symptoms of hepatic encephalopathy, an electroencephalogram is feasible. If necessary, head computed tomography (CT) or MRI can be used to evaluate cerebral edema and exclude cerebrovascular accidents.

### Diagnosis criteria and flow diagram

**Fig. 1:**
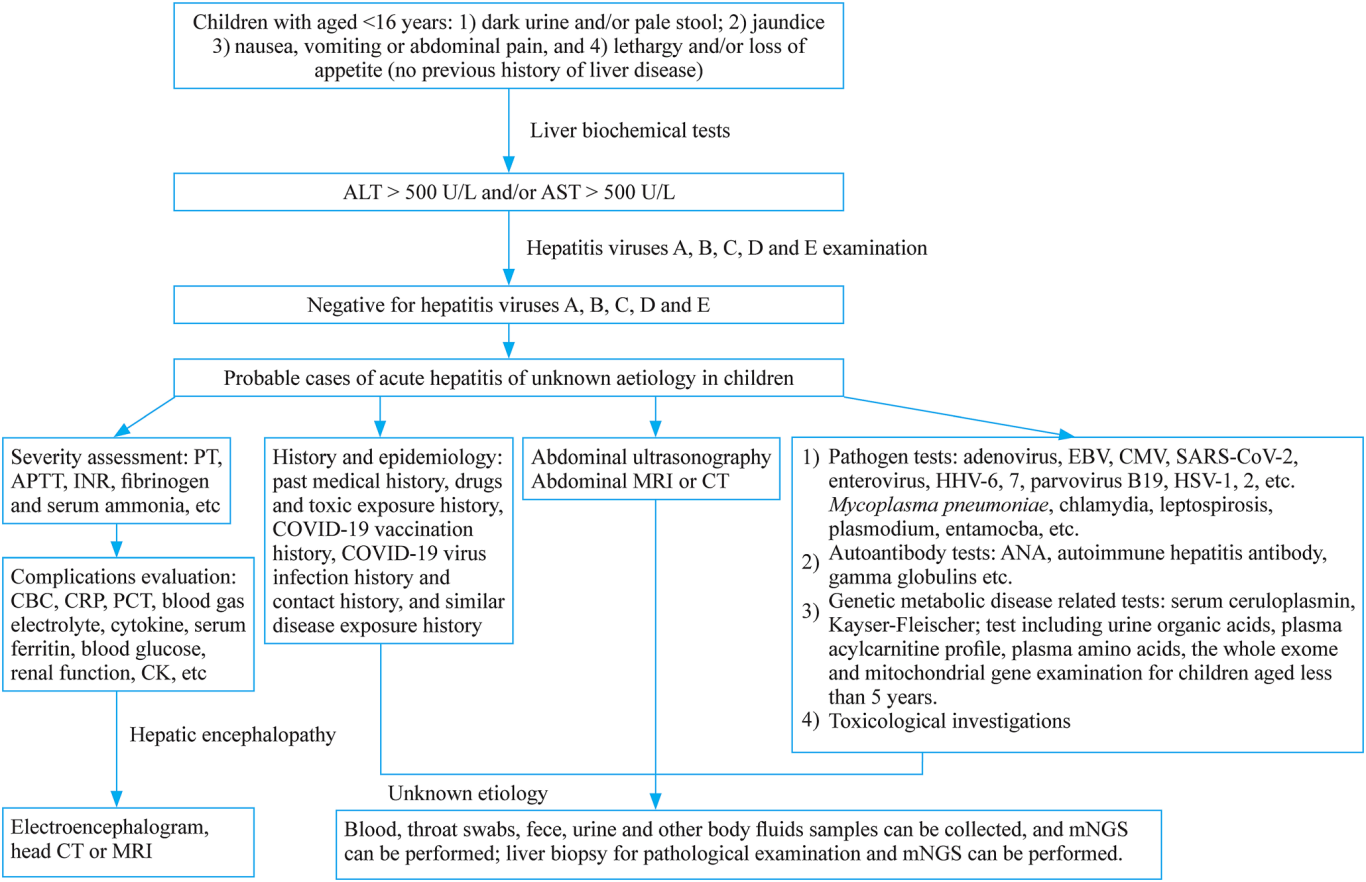
A diagnostic flowchart for reference. *AST* aspartate transaminase, *ALT* alanine transaminase, *PT* prothrombin time, *APTT* activated partial thrombin time, *INR* international standardized ratio, *CBC* complete blood count, *CRP*, c-reactive protein, *PCT* procalcitonin, *CK* creatine kinase, *CT* computer tomography, *MRI* magnetic resonance imaging, *EBV* Epstein–Barr virus, *CMV* cytomegalovirus, *HHV-6* human herpesvirus-6, *HHV-7* human herpesvirus-7, *HSV-1* herpes simplex virus type 1, *HSV-2* herpes simplex virus type 2, *ANA* anti-nuclear antibody

According to the case definition issued by the WHO on 23 April 2022 [4], Table 1 shows diagnostic criteria for acute hepatitis of unknown etiology in children. Fig. 1 shows the diagnostic flow diagram for reference.

**Table 1:** Diagnostic criteria for acute hepatitis of unknown etiology in children [4]

1. Confirmed: not applicable at present;
2. Probable: child with acute hepatitis (non-Hepatitis A, B, C, D, and E virus infection^a^) and serum transaminase (ALT or AST) > 500 IU/L, age ≤ 16 years old, since 1 October 2021
3. Epi-linked: A person of any age with acute hepatitis (non-Hepatitis A, B, C, D and E virus infection^a^) who had close contact with the probable case since 1 October 2021

^a^If a patient is waiting for hepatitis A, B, C, D and E test results but meets other criteria, he can be reported and classified as “pending classification”. Cases with other explanations for hepatitis are ruled out [4]

A diagnostic flowchart is shown in fig 1. AST aspartate transaminase, ALT alanine transaminase, PT prothrombin time, APTT activated partial thrombin time, INR international standardized ratio, CBC complete blood count, CRP, c-reactive protein, PCT procalcitonin, CK creatine kinase, CT computer tomography, MRI magnetic resonance imaging, EBV Epstein–Barr virus, CMV cytomegalovirus, HHV-6 human herpesvirus-6, HHV-7 human herpesvirus-7, HSV-1 herpes simplex virus type 1, HSV-2 herpes simplex virus type 2, ANA anti-nuclear antibody

### Differential diagnosis

(1) *Acute viral hepatitis* Caused by hepatitis A-E virus, can be diagnosed by hepatitis virus serology and/or PCR test.

(2) *Other infectious diseases* ① Viruses: enterovirus, cytomegalovirus, Epstein–Barr virus, herpes simplex virus-1 and 2, human herpes virus-6 and 7, respiratory syncytial virus, parvovirus B19, influenza virus, human immunodeficiency virus, etc. [7, 9]; other pathogens: leptospirosis, plasmodium, amoeba, etc. Identification of pathogens should be based on epidemiological surveys, clinical manifestations and detection of pathogenic organisms.

(3) *Medicine or toxicant-related hepatitis* [18–20] Acetaminophen is the most common cause of drug-induced hepatitis in children. Nonacetaminophen drugs, such as antibiotics, antiepileptic drugs or herbal medicines, are also related to liver damage. A history of exposure to medicine or toxicants is an important clue to diagnose. A test for serum concentrations is helpful for confirmation of identification.

(4) *Autoimmune diseases* Autoimmune hepatitis (AIH) [21]: the characteristics of AIH include elevated serum transaminase, hypergammaglobulinemia, and the presence of autoantibodies. The histological feature of AIH is interfacial hepatitis dominated by infiltration of lymphocytes and plasma cells. Severe cases will quickly progress to cirrhosis and liver failure. Hemophagocytic lymphohistiocytosis (HLH) [22], including familial HLH and secondary HLH. Secondary HLH can be caused by infection, tumors and other factors. In addition to increased transaminases, the diagnosis of HLH includes high persistent fever, blood cytopenia, hepatosplenomegaly, elevated ferritin, coagulation dysfunction and hemophagocytosis in bone marrow.

(5) *Secondary to underlying diseases* Shock, cardiac insufficiency, drug-induced hypotension and sepsis can lead to ischemic liver disease for hepatic hypoperfusion [19]. Ischemic liver disease can also be identified in Budd-Chiari syndrome and hepatic veno-occlusive disease, and imaging examination is helpful for diagnosis.

## Treatment

Information on treatment strategies is still lacking. The following treatment strategy is a comprehensive summary of the current literature combined with our hospital’s experiences in managing children with severe hepatitis.

### Symptomatic and supportive therapy

The mainstay of treatment for severe acute hepatitis of unknown etiology in children is supporting therapy [4], including adequate rest, avoiding excessive protein intake, and preventing complications. Patient consciousness, volume status, urine volume, blood electrolytes, liver function and coagulation function should be closely monitored during the entire treatment period, as should maintaining water, electrolyte and acid–base balance. Subsequent symptoms, such as hypovolemia, hypoproteinemia, gastrointestinal bleeding, infection, electrolyte imbalance, hypoglycemia, and constipation, should be actively treated to prevent serious complications, such as hepatic encephalopathy and hepatorenal syndrome.

### Etiological therapy

To date, the etiology of severe acute hepatitis in children is still under investigation. For those positive for adenovirus infection, supportive care is mainly recommended. Although cidofovir has been reported to be effective against adenovirus in solid organ transplant recipients [23] and children with severe viremia [24], more studies are required to approve the benefits in immunecompetent individuals. Only case reports indicated the efficacy of ribavirin [25] for adenovirus infection.

### Management of coagulation disorders

If acute liver failure (ALF) occurs, various factors, such as decreased coagulation factor synthesis, decreased hepatic thrombopoietin, and secondary bacterial infection, may lead to coagulation disorders [26]. Prothrombin activity (PTA) and INR can be used to evaluate coagulation function in clinical practice. Newer techniques, such as thromboelastography (TEG), are also recommended [27]. Currently, there are no blood transfusion guidelines for children with ALF. Mass transfusion of plasma during treatment will confuse the INR trend of ALF. Therefore, fresh frozen plasma or platelets are not recommended for prophylactic correction of coagulation abnormalities [28]. Vitamin K should be administered when combined with vitamin K deficiency [29]; the cryoprecipitate dosage can be adjusted according to the TEG value to keep fibrinogen in the normal range [30].

### Management of hepatic encephalopathy

#### General treatment

The supine position was recommended, with the head of the bed raised by 20–30 °C, to avoid unnecessary interference. Assessment of hepatic encephalopathy grading should be performed regularly. For children with hepatic encephalopathy of grades III and IV, endotracheal intubation is recommended to protect the airway and control ventilation [31] to ensure effective ventilation and adequate oxygenation. Vasoactive drugs were used to maintain normal mean arterial pressure to ensure effective cerebral perfusion pressure. Mild hypothermia and prophylactic antiepileptic therapy have been used in adults to control cerebral edema, but studies in children are still lacking.

#### Serum ammonia-lowering therapy

Lactulose and lactitol can be taken orally to promote intestinal peristalsis and reduce the absorption of intestinal-derived ammonia and toxins [32]. According to the patient's internal environment, such as electrolytes and acid–base conditions, ammonia-lowering drugs such as arginine can be used, as appropriate [33].

#### Treatment for intracranial hypertension

Hypertonic saline, mannitol and furosemide are the main therapeutic drugs for reducing intracranial pressure. When using hypertonic saline, it is recommended to maintain serum sodium at 145–150 mmol/L [31]. Mannitol is often used to control a sharp rise in intracranial pressure. It is not recommended in renal failure, hypovolemia, serum osmolality > 320 mOsm/L [34], or prophylactic use.

#### Artificial liver support

Artificial liver support is an important therapy for ALF. The artificial liver support systems suitable for children include continuous blood purification, plasma exchange (PE), and molecular adsorption recycling systems. The timing of artificial liver therapy should be determined in combination with the pathophysiological characteristics of the patient’s disease, the principle of the artificial liver model, and treatment goals. When liver failure is complicated by severe hyperbilirubinemia, sepsis, or multiple organ failure, early application can be considered [35]. As a transitional treatment modality before LT, artificial liver therapy is suitable for those patients with INR > 3, accompanied by more than one of the following conditions: hepatic encephalopathy of grade II or above, creatinine value > 3.5 mg/dL, and anuria (< 0.5 mL/kg/h) [36, 37]. PE can be used alone or in combination with other modalities in the treatment of ALF.

### Liver transplantation

Some patients have a poor prognosis, and approximately 6–10% of them need LT [5, 38]. Urgent LT is necessary for children with ALF who experience no improvement or even continuous progress after active comprehensive medical treatment [2, 6, 17, 20]. Unfortunately, there are no well-defined and universally consistent indications for LT in ALF children. The following criteria were adopted by Nadalin S et al. [17, 37]: HE ≥ grade III; INR > 2, or INR > 1.5 and associated with HE; bilirubin > 18 mg/dL; increased tendency to hypoglycemia; decreased liver size monitored by sonography (i.e., liver getting smaller because of necrosis). Irreversible serious neurologic damage, uncontrolled sepsis and uncontrolled multiorgan failure are considered contraindications for LT.

Various transplantation techniques should be applied to shorten the waiting time as much as possible in cases of ALF. Cadaveric donor LT (whole, split or reduced-size), living donor LT and even domino transplantation are applicable LT techniques [39]. With emergency LT, living donor LT may be the only option to save a patient’s life because of the lack of suitable donors [40]. Auxiliary LT can provide emergent support for native liver failure, offering a bridge to the potential spontaneous recovery of the native liver [41].

## Prevention

The etiology of severe acute hepatitis in children is unknown. At present, it is recommended to adopt routine prevention methods for adenovirus and respiratory viruses, such as hand hygiene and respiratory hygiene [4]. Clinicians are asked to be alert to children presenting with symptoms and signs of hepatitis that may require serum transaminase testing and are encouraged to diagnose, investigate and report potential cases [4].

## Conclusions

The outbreak has a global trend of concentrated occurrence without epidemiological links. The etiology is not clear and may be related to adenovirus, but there is no direct evidence. The clinical features are severe acute hepatitis with significantly elevated liver enzymes. The hierarchical treatment strategy is recommended according to the severity of the disease.
